# ﻿Exploring the diversity of *Eupolyphaga* Chopard, 1929 (Blattodea, Corydioidea): species delimitation based on morphology and molecular analysis

**DOI:** 10.3897/zookeys.1120.87483

**Published:** 2022-09-05

**Authors:** Wei Han, Lu Qiu, Jing Zhu, Zong-Qing Wang, Yan-Li Che

**Affiliations:** 1 Institute of Entomology, College of Plant Protection, Southwest University, Beibei, Chongqing 400716, China Southwest University Chongqing China; 2 Engineering Research Center for Forest and Grassland Disaster Prevention and Reduction, Mianyang Normal University, Mianyang 621000, China Mianyang Normal University Mianyang China

**Keywords:** ABGD, Corydiinae, DNA barcoding, new species, Polyphagini

## Abstract

*Eupolyphaga* Chopard, 1929 is a cockroach genus mainly endemic to China. In this study, the species diversity of this genus is further explored through morphology and molecular analysis. Four species are described new to science: *Eupolyphagamiracidia* Qiu, **sp. nov.**, *Eupolyphagaudenostyla* Qiu, **sp. nov.**, *Eupolyphagareducta* Qiu, **sp. nov.**, and *Eupolyphagasimila* Qiu, **sp. nov.** New knowledge on some known species is added, including new distribution records and characteristics of females. Forty-five COI sequences were newly sequenced and a molecular species delimitation analysis was performed using ABGD method. Eighteen molecular operational taxonomic units were obtained by ABGD analysis, which are nearly consistent with the results of morphological delimitation.

## ﻿Introduction

*Eupolyphaga* Chopard, 1929 is the largest genus of Corydioidea in China, including 20 species and two subspecies (Chopard 1929; Feng and Woo 1988; Woo and Feng 1992; Qiu et al. 2018). Sexual dimorphism is pronounced in *Eupolyphaga* species: the male adult is winged with its body narrowly oval, while the female adult is apterous with a sub-oval body. Although this genus is famous for having species with medicinal value, e.g., *E.sinensis* farmed in China as one kind of traditional Chinese medicine (Woo 1987), the taxonomic history of *Eupolyphaga* is rather brief. This genus was established by Chopard (1929) with *Eupolyphagasinensis* as the type species, and a total of five species was included. After this, an additional two species from Yunnan were described (Feng and Woo 1988; Woo and Feng 1992). There was no further taxonomic work on this genus until Qiu et al. (2018), who made a revision to this genus and updated the total number of species to 20.

Species delimitation of the genus has been based on external morphological characters, male genitalia and some oothecae characters (Chopard 1929; Feng and Woo 1988; Woo and Feng 1992; Qiu et al. 2018). Qiu et al. (2018) pointed out that male genitalia played a limited role in species identification of *Eupolyphaga*, because most species do not show very clearly distinct features. As more specimens are collected, boundaries between some species become ambiguous. For example, a new species described below (SWU-B-CC-010023 to SWU-B-CC-010027) collected in Sichuan province is very similar to *E.yunnanensis* in males, while their females and oothecae have significant differences, we therefore consider them to belong to different species.

Automatic Barcode Gap Discovery (ABGD) (Puillandre et al. 2012) is now a common DNA-based delimitation method. So far, it has been successfully applied in many studies about cockroaches (e.g., Bai et al. 2018; Yang et al. 2019; Deng et al. 2020; He et al. 2021; Wang et al. 2021; Zhu et al. 2022), and this method combined with morphological characters can help us judge whether they are different species or not.

We recently obtained more *Eupolyphaga* material from various collecting sites in China. In this study, we use morphological characteristics combined with ABGD to delimit species. Four new species are established. Some descriptions of females and oothecae of known species, and some new distributional data are added to complement species descriptions and facilitate future identifications.

## ﻿Materials and methods

### ﻿Material

Specimens, including the voucher specimens (Table 1) studied in this article, are deposited at the Institute of Entomology, College of Plant Protection, Southwest University, Chongqing, China (**SWU**).

**Table 1.** Samples used in species delimitation.

Species	Abbreviation	GenBank ID	Collecting information	Remark
*E.sinensis*	EupoSineLN	OP215845	Tongnai Village, Fuxin, Liaoning; 31 July 2016; Lu-Yu Wang	nymph
	EupoSineBJ	OP215846	Mt. Xishan, Beijing; 28 April 2015; Bing-Qiang Wang	male
	EupoSineWH	OP215847	Sushansi, Wuhan, Hubei; 16 August 2019; Chen-Liang Li	male
*E.hanae*	EupoHanaSM	OP215848	Mt. Simianshan, Jiangjin, Chongqing; 31 August 2018; Lu Qiu	male
	EupoHanaDG	OP215849	Daguan Town, Dujiangyan, Sichuan; 19 May 2015; Lu Qiu, Jing-Fei Han	male
	EupoHanaSN1	OP215850	Shehong, Suining, Sichuan; 8 May 2016; Lei Wang	male
	EupoHanaSN2	OP215851	ibid	female
	EupoHanaJY	OP215852	Mt. Jinyunshan, Beibei, Chongqing; 22 August 2018; Lu Qiu	male
	EupoHanaGL	OP215853	Jianzhu Township, Gulin, Sichuan; 1 February 2019; Lu Qiu	female
*E.hupingensis*	EupoHupiSM	OP215854	Hupingshan Town, Shimen, Hunan; 21 May 2016; Hao Xu	male
	EupoHupiJZ	OP215855	Jingzhai, Lu,an, Anhui; 2 August 2018; Yu-chen Zheng	male
*E.robusta*	EupoRobu1	OP215856	Maoxian, Sichuan; 6 August 2019; Lu Qiu, Wei Han, Huan-Yu Ren	male, orange abdominal type
	EupoRobu2	OP215857	ibid	female
	EupoRobu3	OP215858	Miancu Village, Maoxian, Sichuan; 7 August 2019; Zong-Qing Wang	male, orange abdominal type
	EupoRobu4	OP215859	Miancu Village, Maoxian, Sichuan; 7 August 2019; Wei Han, Huanyu Ren	female
	EupoRobu5	OP215860	Wenchuan, Sichuan; 7 August 2019; Wei Han, Huanyu Ren	male, orange abdominal type
	EupoRobu6	OP215861	Wenchuan, Sichuan; 7 August 2019; Zongqing Wang	female
	EupoRobu8	OP215862	Wenchuan, Sichuan; 7 August 2019; Wei Han, Huan-Yu Ren	male, black abdominal type
	EupoRobu7	OP215863	Miansi Town, Wenchuan, Sichuan; 29 May 2020; Jian-Yue Qiu	male, black abdominal type
*E.yunnanensis*	EupoYunnCB	OP215864	Zayü, Tibet; 1 August 2014; Weiwei Zhang	female
	EupoYunnCY	OP215865	Zayü, Tibet; 14 August 2015; Lu Qiu	male
	EupoYunnBO	OP215866	Bomê, Tibet; 11 July 2016; Jian-Yue Qiu, Hao Xu	male
	EupoYunnTM2	OP215867	Tongmai Town, Bomê, Tibet; 13 August 2017; HaoXu, Jian-Yue Qiu	male
	EupoYunnTM3	OP215868	Tongmai Town, Bomê, Tibet; 11 August 2017; Hao Xu, Jian-Yue Qiu	male
	EupoYunnTM1	OP215869	Tongmai Town, Bomê, Tibet; 12 August 2017; Jian-Yue Qiu, Hao Xu	male
*E.fengi*	EupoFeng1	OP215870	Mt. Zixishan, Chuxiong, Yunnan; 7 July 2012; Dong Wang	male
	EupoFeng2	OP215871	ibid	male
*E.dongi*	EupoDong1	OP215872	Mt. Gaoligongshan, Baoshan, Yunnan; 13 April 2017; Zhi-Wei Dong	male
	EupoDong2	OP215873	Mt. Gaoligongshan, Baoshan, Yunnan; June 2020; Lu Qiu, Jin-Lin Liu	nymph
*E.wooi*	EupoWooi	OP215874	Mt. Ailaoshan, Xinping, Yunnan; 11 May 2016; Lu Qiu	female
*E.xuorum*	EupoXuor1	OP215875	Caoke Township, Shimian, Sichuan; 25 August 2016; Hao Xu, Jian-Yue Qiu	male
	EupoXuor2	OP215876	ibid	male
*E.daweishana*	EupoDawe	OP215877	Mt. Daweishan, Pinbian, Yunnan; 16 May 2016; Lu Qiu	nymph
*E.miracidia* sp. nov.	EupoMira	OP215878	Maqiao Town, Xiangyang, Hubei; 13 July 2017; Lu Qiu	male
*E.nigrinotum*	EupoNigr1	OP215879	Mt. Jizushan, Bingchuan, Yunnan; 20 February 2016; Hao Xu, Jian-Yue Qiu	male
	EupoNigr3	OP215880	Mt. Jizushan, Bingchuan, Yunnan; 20 February 2016; Hao Xu, Jian-Yue Qiu	female
	EupoNigr2	OP215881	Mt. Jizushan, Bingchuan, Yunnan; 7 June 2019, local	male
*E.pilosa*	EupoPilo	OP215882	Pantiange Township, Weixi, Yunnan; 21 August 2015; Lu Qiu	male
*E.simila* sp. nov.	EupoSimi1	OP215883	Miyaluo Town, Lixian, Sichuan; 6 October 2019; Lu Qiu, Hao Xu, Zhi-Teng Chen	female
	EupoSimi2	OP215884	ibid	nymph
	EupoSimi3	OP215885	ibid	male
*E.reducta* sp. nov.	EupoRedu	OP215886	Wadi Township, Maoxian, Sichuan; 3 October 2019; Hao Xu, Zhi-Teng Chen, Lu Qiu	nymph
*E.udenostyla* sp. nov.	EupoUden1	OP215887	Keku Township, Wenchuan, Sichuan; 7 August 2019; Huan-Yu Ren, Wei Han	female
	EupoUden2	OP215888	ibid	male
	EupoUden3	OP215889	ibid	male
**Outgroup**				
*Periplanetaamericana*	PeriAmer	HM386405	/	
*Eucorydiadasytoides*	EucuDasy	LC480880	/	
*Diplopterapunctata*	DiplPunc	MF479156	/	

### ﻿Morphology

The morphological terminology follows Roth (2003), Klass (1997) and Li et al. (2018). Male genital segments of the examined specimens were macerated and heated in 10% NaOH solution for ~ 20 min to remove excess fat, then washed with deionized water, placed in glycerin jelly, and observed with a Motic K400 or Leica® M205A stereomicroscope. Habitus photographs of the specimens were taken using a Canon® EOS M5 digital camera+ mount adapter EF-EOS M plus a Laowa 100 mm F2.8 CA-Dreamer Macro 2 × lens (for Canon EF). Photographs of other characters were taken using a Leica® M205A stereomicroscope. All photographs mentioned above were optimized with Adobe Photoshop® CC 2019.

### ﻿DNA extraction, PCR, and sequencing

The hind legs or thoracic muscle were used for DNA extraction, and the other body parts were stored in 95% ethanol as voucher specimens. The extraction procedure was performed according to the Hipure Tissue DNA Mini Kit (Magen Bio-tech, Guangzhou) and the extracted total DNA was stored in a -20 °C environment. Fragments of COI were amplified using PCR; primers used for the amplifications are F: 5’-GGTCAACAAATCATAAAGATATTGG-3’ and R: 5’-TAAACTTCAGGGTGACCAAAAAATCA-3’ (Folmer et al. 1994). PCR was performed in an Analytik Jena Easy Cycler with 25 μl volumes including 22 μL 3×Taq Master Mix, 1 μL of each primer (F and R, aforementioned), and 1 μL of DNA template. Amplification conditions were: initial denaturation at 98 °C for 2 min, followed by 35 cycles for 10 s at 98 °C, 10 s at 50 °C, 10 s at 72 °C, and a final extension of 5 min at 72 °C. Then the amplification effect was assessed by electrophoresis in 1% agarose gel, the amplification products corresponding to bright and clear bands were selected and sent to the Tsingke Biotechnology Co., Ltd. (Beijing, China) for sequencing in both directions.

### ﻿Sequence processing and phylogenetic analyses

In total, 48 COI sequences were used for analysis (45 sequences representing *Eupolyphaga* species and three sequences representing outgroups downloaded from GenBank). All 45 newly acquired sequences are deposited in GenBank (https://www.ncbi.nlm.nih.gov/nuccore) with accession numbers OP215845 to OP215889 (Table 1). Sequences were aligned by online MAFFT 7 (https://mafft.cbrc.jp/alignment/server/) (Katoh et al. 2019) using the Q-INS-i algorithm, then aligned and adjusted manually after translation into amino acid sequences using MEGA 11 (Kumar et al. 2016). A Kimura 2-parameter (Kimura 1980) distance model was used to quantify intraspecific and interspecific genetic divergence values. The maximum likelihood tree was constructed in IQ-TREE (Nguyen et al. 2015) with 1000 replicates for bootstrap values, after choosing optimal partitioning scheme and substitution models (COI_pos 1, TRN+G; COI_pos 2, GTR+G; COI_pos 3, GTR+G) in PartionFinder v.2.1.1 (Lanfear et al. 2017) with the corrected Akaike Information Criterion (AICc).

We also conducted an ABGD analysis to confront its results to our morphologically defined species and thus further refine our species delimitation within the genus *Eupolyphaga*. ABGD analysis was performed using a web interface (https://bioinfo.mnhn.fr/abi/public/abgd/abgdweb.html); the default parameters were used except for the relative gap width set at 1.0 and using the Jukes-Cantor (JC69) model.

## ﻿Results

### ﻿Morphological delimitation of *Eupolyphaga*

**Figure 1.**
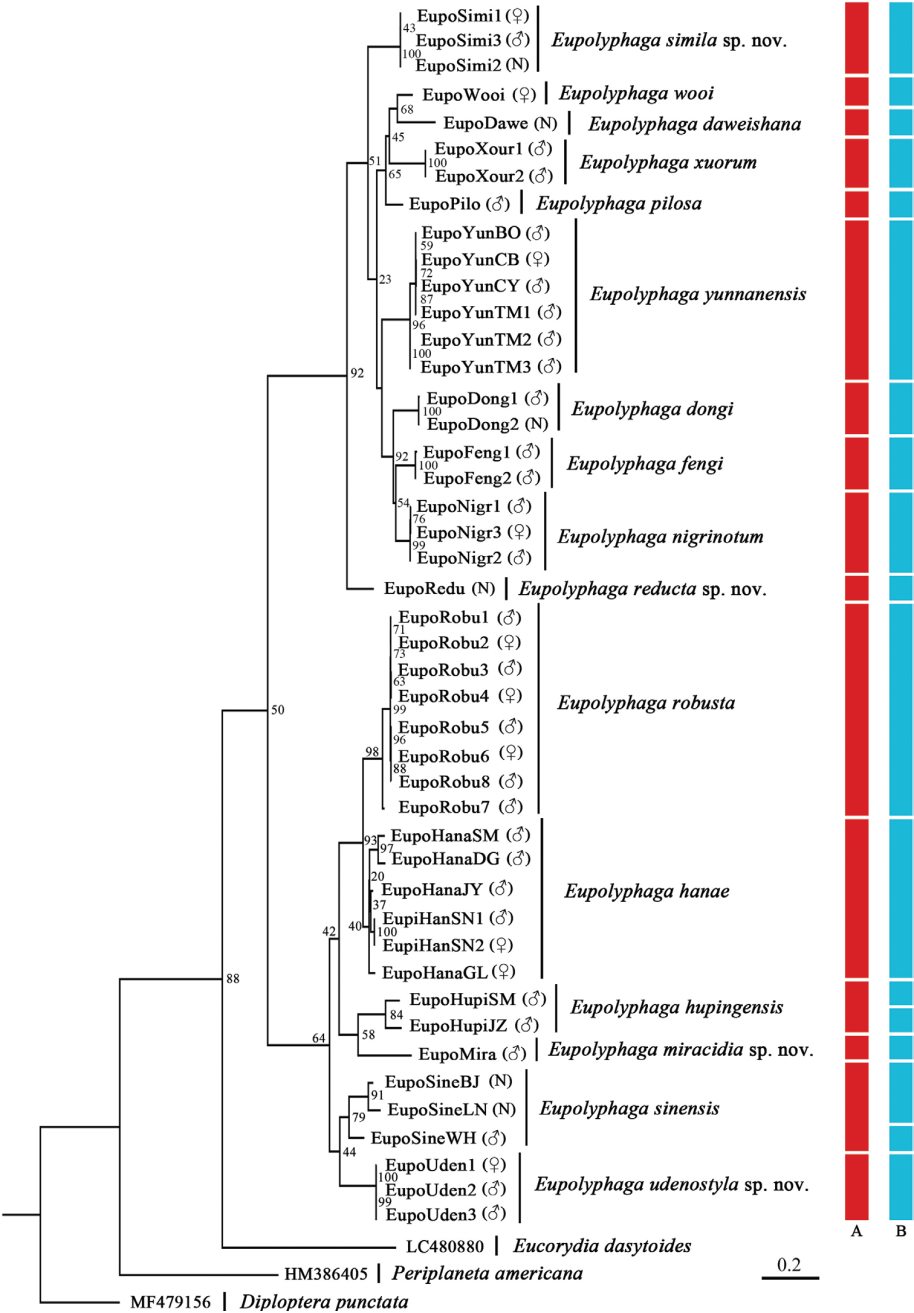
Maximum Likelihood tree derived from COI sequences with 1000 bootstrap replicates. Bootstrap values are marked at nodes, the sexes of the specimens are given in brackets (“N” indicates nymphs). Red bar: results of morphological delimitation; blue bar: results of MOTUs estimated by ABGD.

Combining male and female external morphology, male genitalia, and some oothecae features, we identified 16 morphospecies (including four new species) of *Eupolyphaga* among 266 samples examined from China (Fig. 1A).

### ﻿Phylogenetic analysis and MOTUs estimation based on COI

In this study, we acquired 45 COI sequences of *Eupolyphaga* representing 16 morphospecies of *Eupolyphaga*. The maximum likelihood tree shows that, for each morphological species, all samples (including the different locality samples, different sex samples, and adult and nymphal samples) are monophyletic, although most of the nodes do not have high bootstrap values (Fig. 1).

Eighteen molecular operational taxonomic units (**MOTUs)** were estimated by ABGD for the 45 samples (Fig. 1B), and 14 morphological species were well supported by the ABGD result; morphological assumptions and molecular results differ only for *E.sinensis and E.hupingensis*. The three samples of *E.sinensis* collected from three different localities (Beijing, Liaoning, and Wuhan) are estimated as two MOTUs. The two samples of *E.hupingensis*, collected from Hunan and Anhui provinces, were also divided into two MOTUs.

### ﻿Systematics

#### ﻿ Eupolyphaga miracidia

Taxon classificationAnimaliaBlattodeaCorydiidae

Qiu sp. nov.

B312FF13-B2C2-5676-BF01-05AF8F2DF50C

https://zoobank.org/90674A34-2596-438B-9668-5CEAA24103EE

Fig. 2

##### Type material.

***Holotype***: China · male; Hubei Prov., Xiangyang City, Maqiao Town, roadside of Ganxigou; 480 m–600 m; 31°46.99'N, 110°55.06'E; 13 July 2017; Lu Qiu leg.; SWU-B-CC-010001.

***Paratypes***: China · 2 males & 2 females, same collection data as holotype; SWU-B-CC-010002 to SWU-B-CC-010005.

##### Diagnosis.

**Figure 2.**
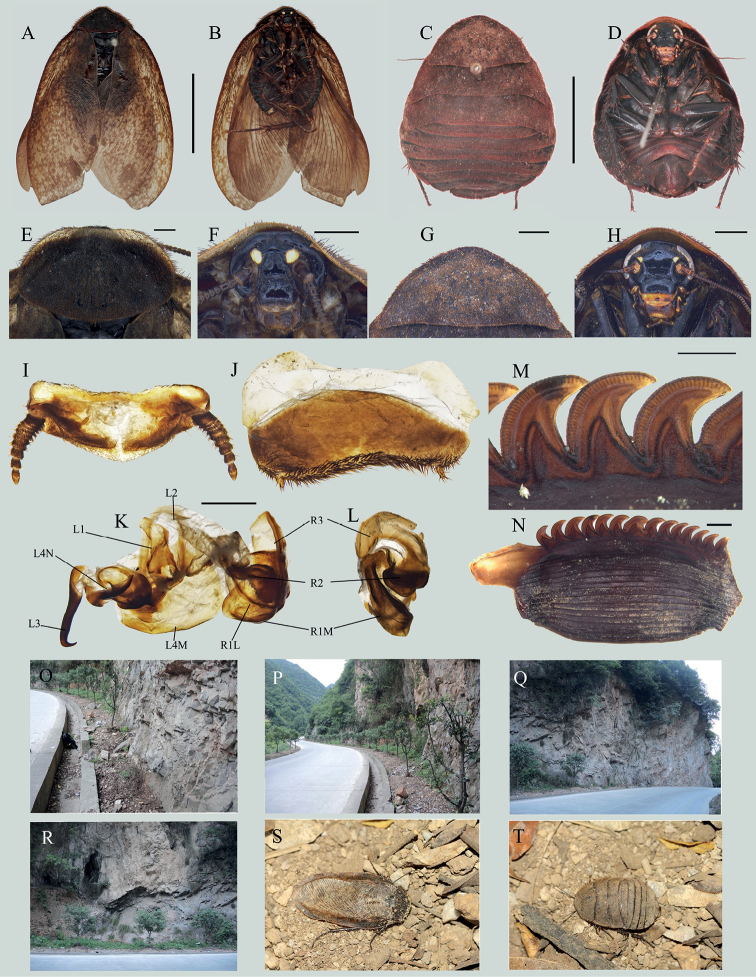
*Eupolyphagamiracidia* Qiu, sp. nov. **A** male holotype, dorsal view **B** male holotype, ventral view **C** female paratype, dorsal view **D** female paratype, ventral view **E** pronotum, male holotype, dorsal view **F** head, male holotype, ventral view **G** pronotum, female paratype, dorsal view **H** head, female paratype, ventral view **I** supra-anal plate, male holotype, ventral view **J** subgenital plate, male holotype, ventral view **K** genitalia, male holotype, dorsal view **L** right phallomere, holotype, right-ventral view **M** the serration of oothecae **N** oothecae, lateral view **O–R** habitats of *E.miracidia* Qiu, sp. nov., in Maqiao Town, Xiangyang City, Hubei Province **S** living male **T** living female. Scale bars: 1.0 cm (**A–D**); 0.1 cm (**E–L, N**); 0.02 cm (**M**). Photographs **O–T** by LQ.

This species can be easily distinguished from other species by its small body size. It resembles *E.hupingensis* by its dark coloration and dense maculae on tegmina, but it can be distinguished from the latter by its small-sized body in both sexes (12.1–12.5 mm excluding tegmina and wings in males, 22.3–24.1 mm in females; Fig. 2A–D), small styli (Fig. 2J), slenderer genital hook (Fig. 2K), and the relatively separated spaces between the serrations of the ootheca (Fig. 2M, N).

##### Description.

**Male holotype. *Measurements* (mm).** Overall length: 26.9; body length: 12.1; body width (tegmina not included): 9.8; tegmen length × width: 22.3 × 9.5; pronotum length × width: 7.4 × 4.4.

***Coloration*.** Body blackish brown, covered with brown setae (Fig. 2A, B). Head black, ante-clypeus whitish, sub-transparent. Antenna brown. Pronotum dark-brown, anterior margin yellowish white. Tegmina sub-transparent, yellowish brown, with dark brown maculae. Wing light brown, with darkish brown maculae. Leg blackish brown, with coxa and trochanter slightly tawny. Abdomen blackish brown, slightly yellowish brown medially.

***Head***: Subrounded, almost hidden under pronotum. Interocular space moderate, about half of the distance between ocelli. Ocelli large, ocelli ridge protruded and curved, two dimples symmetrically situated below the ridge. Two yellowish brown semicircular pits each situated next to the inner side of antennal sockets. Clypeus distinct, labrum almost quadrate, posterior margin concave in the middle (Fig. 2F). ***Pronotum***: Small, widest near the middle, anterior whitish margin narrow, gradually narrowing from the middle to lateral sides, boundary between the white and dark brown areas distinct (Fig. 2E). ***Tegmina and hind wings***: Extending beyond the end of abdomen 12.1 mm, maculae on tegmina dense, fused. ***Legs***: Slender, front femur type C_1_, pulvilli moderate and tarsal claws simple, symmetrical, arolia large. ***Abdomen***: Supra-anal plate transverse, pubescent, posterior margin prominent medially, paraprocts simple, cerci short (Fig. 2I). Subgenital plate asymmetrical, hind margin concave medially and densely setose, left side less prominent than right side. Styli small, the left one longer than the right one (Fig. 2J). ***Genitalia***: L1 basally prolonged (Fig. 2K), genital hook (L3) slender, the hooked part roundly curved, apex sharp (Fig. 2K). Right phallomere small, R2 simple, broad, and concave in the middle (Fig. 2L).

**Male paratypes.** Similar to the holotype, no distinct differences.

**Female paratypes.** Body length: 22.3–24.1 mm, body width: 16.2–16.9 mm. Body uniformly dark reddish brown (Fig. 2C, D). Antennal sockets and ocelli pale yellow, ante-clypeus yellow. Labrum yellowish white, base and ends slightly lighter in color. Ocelli moderate in size, nearly triangular. Interocular space almost equal to distance between antennal sockets, and bigger than the distance between ocelli (Fig. 2H). Arolia and pullivi absent. Posterior margin of the supra-anal plate emarginated medially.

**Nymph.** Similar to the female, body darkish brown.

**Ootheca.** Reddish brown. The longitudinal lines distinct. Serrations on the keel large and curved, apex of serrations slightly truncated. Space between the serrations of the curved portion moderate (Fig. 2M). Respiratory canals well developed.

##### Natural history.

Found in dry earth under a cliff of the roadside (Fig. 2O–R).

##### Etymology.

The species epithet *miracidia* is derived from Greek *mirakos* referring to it's small size.

#### ﻿ Eupolyphaga udenostyla

Taxon classificationAnimaliaBlattodeaCorydiidae

Qiu sp. nov.

1A42FF71-585E-5E44-AFAB-0BFC89F1BB4C

https://zoobank.org/ED205416-E3D8-4955-AE89-7D50DEB363DE

Figs 3 , 4B, D, E–J

##### Type material.

***Holotype***: China · male; Sichuan Prov., Aba Prefecture, Wenchuan County, Keku Township; 1555 m; 31°30.93'N, 103°34.27'E, 5 May 2019; Lu Qiu leg.; SWU-B-CC-010006.

***Paratypes***: China · 3 males & 1 female, same collection data as holotype; SWU-B-CC-010007 to 010010 · 3 males, same collection data as holotype, but 7 August 2019; Huan-Yu Ren, Wei Han leg; SWU-B-CC-010011 to 010013 · 2 males & 4 females, Sichuan Prov., Wenchuan County, mountains behind the 5·12 Wenchuan Earthquake Memorial Museum; July–August 2019; Qi Li leg.; SWU-B-CC-010014 to 010019.

##### Diagnosis.

This species is remarkable for the absence of styli and the short tegmina and strongly reduced anal fields of hind wings in male, which can easily distinguish the males of *E.udenostyla* from all other species. The serrations of ootheca are distinctly reduced.

##### Description.

**Male holotype**: Body stout. ***Measurements* (mm).** Overall length: 23.5; body length: 19.7; body width (tegmina not included): 12.1; pronotum length × width: 17.1 × 9.4; tegmina length × width: 17.6 × 8.4.

**Figure 3.**
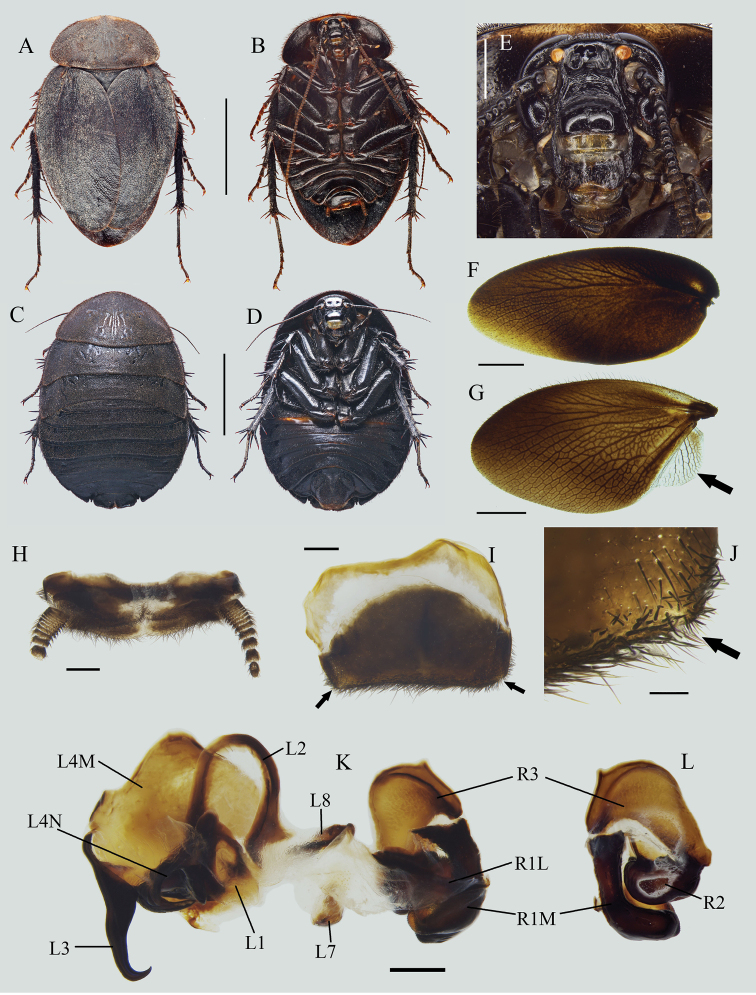
*Eupolyphagaudenostyla* Qiu, sp. nov. **A** male holotype, dorsal view **B** male holotype, ventral view **C** female paratype, dorsal view **D** female paratype, ventral view **E** male head, ventral view **F** tegmen, ventral view **G** hind wing, ventral view **H** supra-anal plate, ventral view **I** subgenital plate, ventral view **J** left portion of subgenital plate, dorsal view **K** genitalia, dorsal view **L** right phallomere, right-ventral view. Scale bars: 1.0 cm (**A–D**); 0.2 cm **E**; 0.5 cm (**F, G**); 0.1 cm (**H, I, K, L**); 0.02 cm (**J**). Black arrows indicate the strongly reduced anal field and absence of styli.

***Coloration*.** Body almost black, covered with black setae (Fig. 3A, B). Anterior margin of pronotum partially white (Fig. 3A).

**Figure 4.**
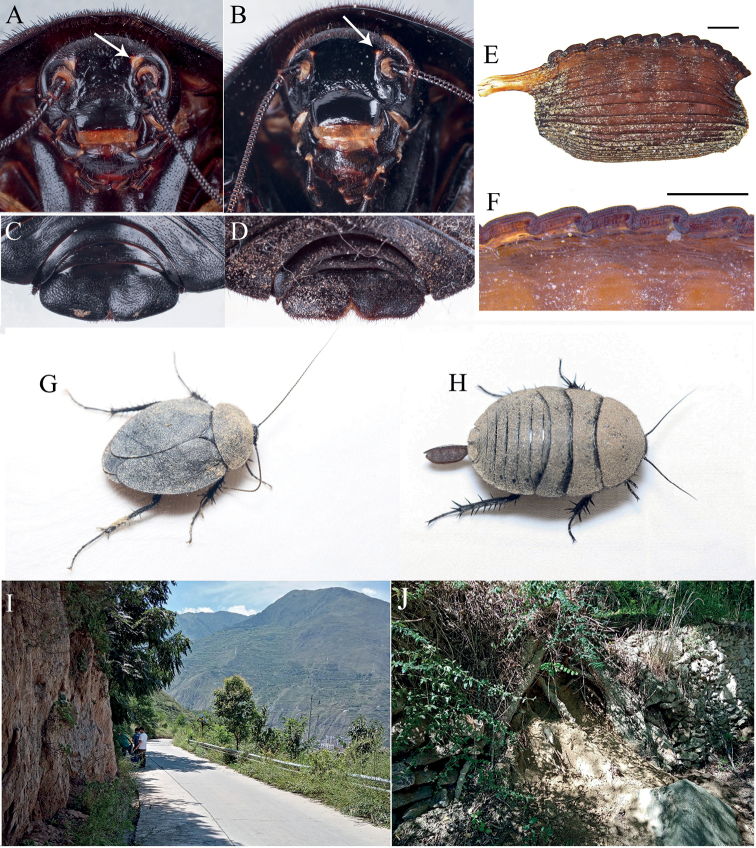
**A–D** Characteristics and habitats of *Eupolyphaga* species from Minjiang River Basin **A** head of female *E.robusta*, ventral view **B** head of female *Eupolyphagaudenostyla* Qiu, sp. nov., ventral view **C** supra-anal plate of female *E.robusta*, dorsal view **D** supra-anal plate of female *E.udenostyla* Qiu, sp. nov., dorsal view **E** ootheca of *E.udenostyla* Qiu, sp. nov., lateral view **F** same, close-up view to show the serration **G** a living male *E.udenostyla* Qiu, sp. nov. **H** a living female *E.udenostyla* Qiu, sp. nov. **I, J** habitats of *E.udenostyla* Qiu, sp. nov., in Keku Township, Wenchuan County, Sichuan Province. Scale bars: 0.2 cm (**E**); 0.1 cm (**F**). Photographs **G–J** by LQ. White arrows indicate the ocelli.

***Head***: Ovoid, longer than wide. Vertex concealed under pronotum. Eyes small, slightly reduced. Interocular space wide but narrower than the distances between antennal sockets and between ocelli. Ocelli moderate, ocellar ridge distinct, with a row of setae on the upper edge (Fig. 3E). Antennae long, approximately equal to the length of the body (vertex to abdominal tip) (Fig. 4G). Ocellar ridge with two large pits below. Clypeus distinct, ante-clypeus almost quadrate, hind lateral angles obtusely rounded, posterior margin thin in the middle and slightly concave. ***Pronotum***: Narrow, widest near the middle, anterior whitish margin narrow and short. ***Tegmina and hind wings***: Shortened, only beyond the end of abdomen 3.8 mm. Apices of wings slightly exceed tegmina in resting position. Tegmen rounded apically (Fig. 3F). Hind wing short, oval, bluntly rounded apically, anal field strongly reduced (Fig. 3G). ***Legs***: Slender, front femur type C_1_. Pulvilli moderate, tarsal claws simple and symmetrical, arolia large. ***Abdomen***: Supra-anal plate narrowly transverse, pubescent, hind margin with an emargination medially, paraprocts simple, cerci short (Fig. 3H). Subgenital plate simple, posterior margin nearly straight, densely setose. Styli absent (Fig. 3I, J). ***Genitalia***: L1 narrowed at base, genital hook (L3) stout basally, gradually tapered at distal half, the hook roundly curved, apex sharp (Fig. 3K).

**Male paratypes**: Similar to the holotype, no significant variation.

**Female paratypes**: Body length: 23.5–24.7 mm, body width: 17.5–18.1 mm. Body uniformly dark brown (Fig. 3C, D), ante-clypeus and base of labrum white. Eyes and ocelli small, with a pair of large dimples between antennal sockets (Fig. 3D). Distance between ocelli smaller than distance antennal sockets, and both smaller than interocular space (Fig. 4B). Arolia and pulvilli absent. Supra-anal plate straight at posterior margin, lateral portions somewhat angular, posterior margin with a wide hollow medially (Fig. 4D).

**Nymph.** Similar to the female, body uniformly black or dark brown.

**Ootheca.** Reddish brown. Serrations of keel small, blunt. Surface with elevated longitudinal lines, the width of ridge wider near the serrations (Fig. 4E, F).

##### Natural history.

This new species is distributed in the Minjiang River Basin (Wenchuan County) (Fig. 4I, J). Individuals hide in the soil and are active during night (LQ, pers. obs.).

##### Etymology.

*uden*- (Greek) + *styla* (Greek) indicate that the males of the new species have no styli.

##### Remarks.

The absence of styli in males makes this new species morphologically remarkable in *Eupolyphaga*. Males of the species also have distinctly shortened tegmina and wings, and extremely reduced anal areas on hind wings. The ootheca has reduced serrations. The type locality of the new species is close to the one of *Eupolyphagarobusta*, they are sympatric, distributed in Minjiang River Basin. Males and ootheca of this new species are easily distinguished from *E.robusta*, but females may be confused because of their similar black and large body. However, females of this new species have reduced ocelli (Fig. 4B), which are obviously smaller than those of *E.robusta* (Fig. 4A). Also, the posterior margin of the supra-anal plate (Fig. 4D) in this new species is obviously straighter than that of *E.robusta* (Fig. 4C, D).

#### ﻿ Eupolyphaga reducta

Taxon classificationAnimaliaBlattodeaCorydiidae

Qiu sp. nov.

F8FBBA76-49D5-535C-A37C-DFDED842D126

https://zoobank.org/8749380B-3F77-4033-90D3-69A43F905ADD

Fig. 5

##### Type material.

***Holotype***: China · male, Sichuan Prov., Maoxian County, Wadi Township, Shahuzhai Village; 1938 m; 31°53.20'N, 103°28.62'E; 3 October 2019; Hao Xu, Zhi-Teng Chen, Lu Qiu leg (the holotype was reared from nymph by LQ).; SWU-B-CC-010020.

##### Other specimens examined.

1 male (an incompletely eclosed individual, unsuitable to be designated as a type specimen); SWU-B-CC-010021 · 1 female nymph, same collection data as holotype; SWU-B-CC-010022.

##### Diagnosis.

This species is easily distinguished from its congeners by the reduced and stout tegmina and hind wings in male. In comparison with other species also possessing reduced tegmina and hind wings in males (e.g., *E.everestianareni* and *E.udenostyla* Qiu, sp. nov.), this new species is obviously larger (24.7 mm without tegmina and wings in length), the tegmina with sparse macula only on distal half; while *E.everestianareni* is small in size (18.0–18.5 mm without tegmina and hind wings in length) and with evenly distributed maculae on tegmina. *E.udenostyla* Qiu, sp. nov. (19.7 mm without tegmina and wings in length) is an almost unicolored black species with no styli in males.

##### Description.

**Figure 5.**
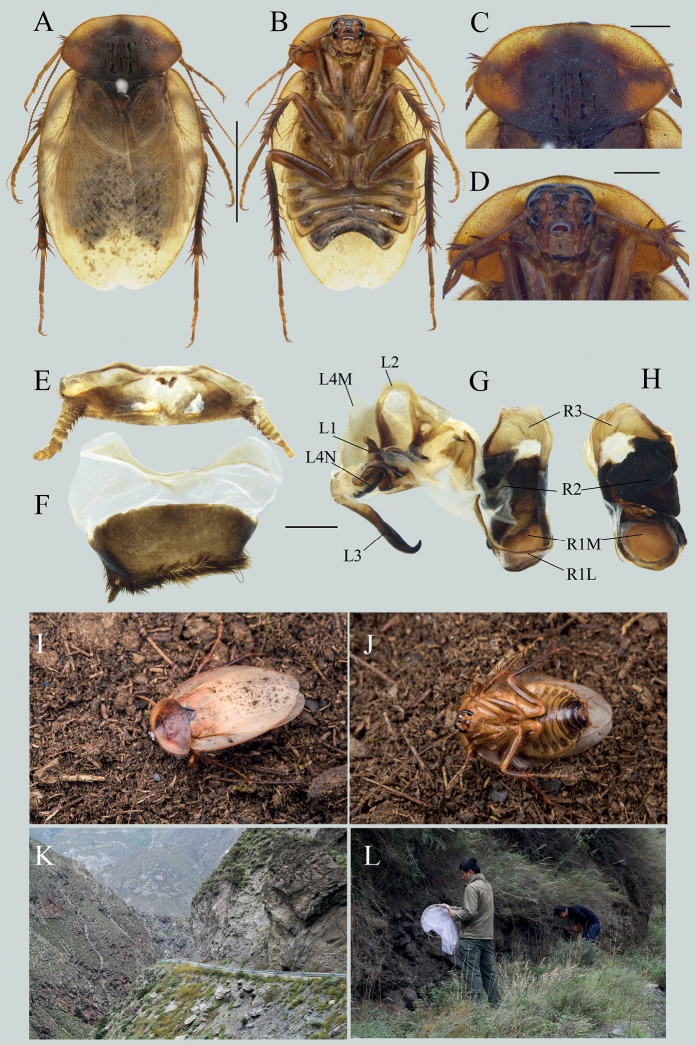
*Eupolyphagareducta* Qiu, sp. nov., male holotype **A** habitus, dorsal view **B** habitus, ventral view **C** pronotum, male holotype, dorsal view **D** head, male holotype, ventral view **E** supra-anal plate, ventral view **F** subgenital plate, ventral view **G** genitalia, dorsal view **H** right phallomere, right-ventral view **I–J** the living holotype after moulting **K** habitat of *E.reducta* Qiu, sp. nov., in Shahuzhai Village, Maoxian County, Sichuan Province **L** Dr. Zhi-Teng Chen and Dr. Hao Xu collecting this species. Scale bars: 1.0 cm (**A, B**); 0.2 cm (**C, D**); 0.1 cm (**E–H**). Photographs **I–L** by LQ.

**Male holotype**: Body short and broad (Fig. 5A, B). ***Measurements* (mm)**: Overall length: 29.5; body length: 24.7; body width (tegmina not included): 13.4; pronotum length × width: 12.5 × 6.9; tegmina length × width: 22.5 × 9.3.

***Coloration*.** Body yellowish brown. Pronotum brown, anterior margin yellow (Fig. 5C). Space between vertex to the ocelli black, face fulvous with blackish brown spots. Antennae yellowish brown, ante-clypeus yellow, hind-clypeus brown. Labial palpi and maxillary palpi yellowish brown (Fig. 5D). Tegmina and hind wings yellowish brown, maculae brown. Legs and abdomen brown.

***Head***: Interocular space narrow, obviously narrower than the distance between antennal sockets, and slightly narrower than the distance between ocelli. Ocelli large, ocellar ridge short, straight, covered with yellowish brown setae. Clypeus small (Fig. 5D). ***Pronotum***: Widely transverse, the widest point near the middle, anterior yellowish margin wide, the boundary between the yellowish and the brownish portion fused. Surface with short pubescence (Fig. 5C). ***Tegmina and hind wings***: Reduced and robust, exceeding the end of abdomen 4.4 mm. The apices of hind wings exceed tegmina obviously when at resting position (Fig. 5I). Tegmina translucent, yellowish brown, irregularly with several maculae apically, shoulders of tegmina wide, margins rounded. Wings translucent, light yellowish brown, almost no maculae. ***Legs***: Slender, front femur type C_1_, arolia large, tarsal claws simple and symmetrical. ***Abdomen***: Supra-anal plate transversely broad, convex in the middle, cerci short (Fig. 5E). Subgenital plate slightly asymmetrical, right portion larger than left, styli short (Fig. 5F). ***Genitalia***: Well sclerotized (Fig. 5G, H). L1 slender, anteriorly prominently protruded, with a long protuberance on the left side and two well-developed protrusions on the posterior margin, L2 more developed on the right side, L3 curved, with a more rounded curvature, processa distale (pda) and processa apicale (paa) well developed, long and sturdy. Right phallomere relatively larger, R2 divided into two chunks, outer chunk larger than the inner one.

**Female and ootheca**: Unknown.

**Nymph**: Similar to the male in color, yellowish brown.

##### Natural history.

This new species is distributed in the Heishui River Basin (Maoxian County to Heishui County). Nymphs of this new species live beneath the withered grass at the edge of a soil slope (Hao Xu, Lu Qiu, pers. obs.; Fig. 5K, L).

##### Etymology.

The species epithet is from the Latin *reductus* indicating its reduced tegmina and wings.

#### ﻿ Eupolyphaga simila

Taxon classificationAnimaliaBlattodeaCorydiidae

Qiu sp. nov.

7B890669-E799-502C-BF1B-53E97F41CBFB

https://zoobank.org/903D40EF-A9B2-49A7-9296-A5749B3D6D9D

Figs 6 , 7

##### Type material.

***Holotype***: China · male; Sichuan Prov., Lixian County, Miyaluo Town, Siboguo Village; 2944 m; 31°41.58'N, 102°44.80'E; 6 October 2019; Hao Xu, Lu Qiu, Zhi-Teng Chen leg.; SWU-B-CC-010023.

***Paratypes***: 2 males, same collection data as holotype; SWU-B-CC-010024 to 010025.

##### Other material examined.

2 nymphs & 4 oothecae, same collection data as holotype; SWU-B-CC-010026 to 010027.

##### Diagnosis.

**Figure 6.**
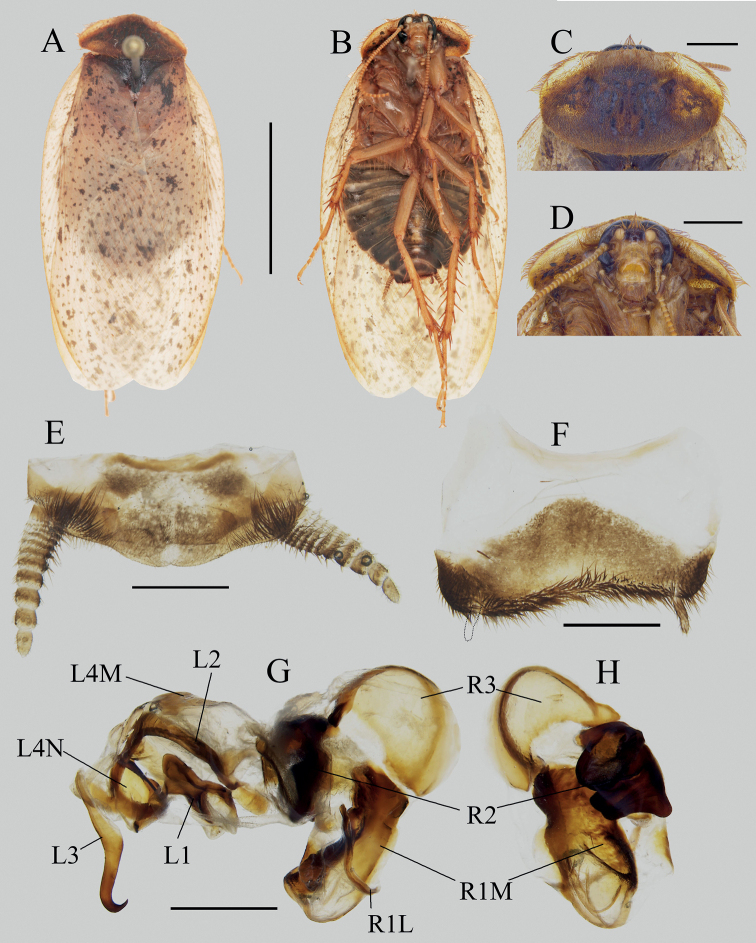
*Eupolyphagasimila* Qiu, sp. nov., male holotype **A** habitus, dorsal view **B** habitus, ventral view **C** pronotum, dorsal view **D** head, ventral view **E** supra-anal plate, ventral view **F** subgenital plate (right stylus missing), ventral view **G** genitalia, dorsal view **H** right phallomere, right-ventral view. Scale bars: 1.0 cm (**A, B**); 0.2 cm (**C, D**); 0.1 cm (**E–H**).

**Figure 7.**
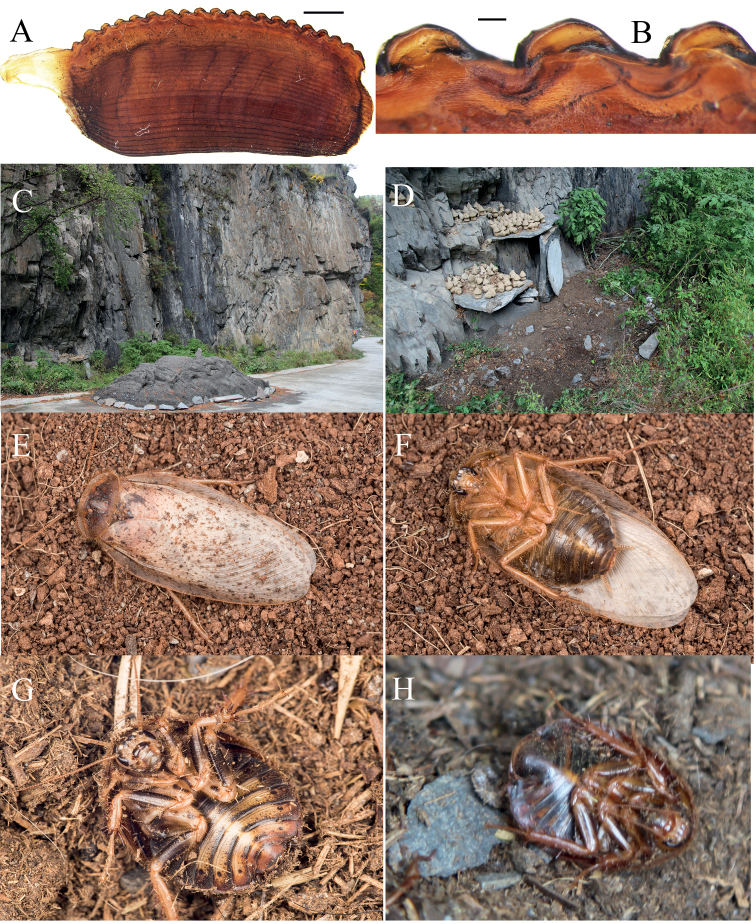
*Eupolyphagasimila* Qiu, sp. nov. **A** ootheca, lateral view **B** serrations of ootheca **C, D** habitats of *Eupolyphagasimila*, Siboguo Village, Lixian County, Sichuan Province **E, F** the living holotype after emergence **G** the ventral side of a nymph **H** the ventral side of a female. Scale bars: 0.1 cm (**A**); 0.01 cm (**B**). Photographs **C–H** by LQ.

The male of this new species resembles *Eupolyphagayunnanensis* in external morphology. However, the male of *E.simila* Qiu sp. nov. has a darker abdomen (Fig. 6B) than *E.yunnanensis*. The female and nymph of this new species have dense black markings on their bodies (Fig. 7G, H), while *E.yunnanensis* is without black markings. The serrations of ootheca are bluntly rounded in this new species (Fig. 7A, B) while they are triangular in *E.yunnanensis* (Qiu et al. 2018).

##### Description.

**Male holotype: Measurements (mm).** Overall length: 28.9; body length: 16.2; body width (tegmina not included): 9.4; pronotum length × width: 8.3 × 5.1; tegmina length × width: 26.3 × 9.0.

***Coloration*.** Body fulvous (Fig. 6A, B). Vertex to the space between ocelli blackish brown, ocellar ridge slightly lighter in color, the rest of the face yellow. Antennae and labrum yellow-brown (Fig. 6D). Pronotum brown, except for the yellow anterior margin (Fig. 6C). Tegmina fawn with scattered and unequal-sized maculae. Wings nearly transparent, with sparsely small maculae terminally. Legs fulvous. Abdomen darkish brown, with a yellow vertical line in the middle (Fig. 6A, B).

***Head***. Round, longer than wide. Interocular space narrower than the distances between antennal sockets and ocelli. Ocelli bulged, ocellar ridge pubescent (Fig. 6D). ***Pronotum*.** Transversely elliptical, with anterior margin raised medially, and slightly truncated, the widest point located near the middle, and the posterior margin slightly arched. Boundary between anterior yellowish margin and the brownish portion fused (Fig. 6C). ***Tegmina and hind wings*.** Tegmina slender, beyond the end of abdomen 13 mm. Wings transparent. ***Legs*.** Slender and hairy, arolia large. ***Abdomen*.** Posterior margin of supra-anal plate protruded posteriorly, with a median shallow concavity (Fig. 6E). Subgenital plate slightly asymmetrical, media widely concaved, styli long (Fig. 6F). ***Genitalia*.** Genital hook (L3) roundly curved, with a sharp apex. R2 divided into two asymmetrical chunks, slightly quadrate (Fig. 6G, H).

**Male paratype**: Body length 29.1 mm including tegmina and hind wings, no significant differences from the holotype.

**Nymph.** Yellowish brown, dorsal surface black-brown to pale yellow-brown, with many black maculae. Vertex with a black strip between eyes, the space between ocelli with some black spots. Hind-clypeus black. Legs darkish brown to light yellowish brown, coxa with some black maculae. Abdomen yellow, with dark margins (Fig. 7G).

**Ootheca.** Light reddish brown. Surface with longitudinal lines. Serrations of the keel small and bluntly rounded. Respiratory canals absent (Fig. 7A, B).

##### Natural history.

This new species can be found in dry soil under cliffs along the roadside (Fig. 7C, D).

##### Etymology.

The species epithet is from *similis* (Latin) indicating this species is similar to *Eupolyphagayunnanensis*.

##### Remarks.

Due to the outbreak of COVID-19, all living females of the new species were uncared-for in the lab during the research time. Thus, no female specimens survived for further study, but LQ has taken a photograph of a female in the wild (Fig. 7H), which provides a chance to compare the coloration with the female of *E.yunnanensis*. We also distinguished *E.simila* Qiu sp. nov. from *E.yunnanensis* by using DNA barcoding; the genetic divergence of the two species being 13.86–14.82%, and the ABGD analysis also supports the establishment of this new species.

#### ﻿ Eupolyphaga sinensis

Taxon classificationAnimaliaBlattodeaCorydiidae

(Walker, 1868)

7FCC4F0C-3B73-5871-8AEC-1ED6FC64734D

Fig. 8

Polyphaga sinensis Walker, 1868: 14.
Homoeogamia sinensis Saussure, 1869: 282; Hollier et al. 2020: 347. Synonymized by Qiu et al. (2018).
Heterogamia sinensis : Dohrn 1888: 132.
Heterogamia dohrniana Saussure, 1893: 309; Hollier et al. 2020: 345.
Polyphaga limbata Kirby, 1903: 379.
Eupolyphaga sinensis : Chopard 1929: 262; Qiu et al. 2018: 5 (revision); Qiu et al. 2019: 11 (checklist).

##### Type locality.

“North China”.

##### New material examined.

China · 2 males; Hubei Prov., Xiangyang City, Xianshan Mountain; July 2020; Mao Ye leg; SWU-B-CC-010028 to 010029 · 1 male; Hubei Prov., Wuhan City, Huangling District, Sushan Temple; 16 August 2019; Chen-Liang Li leg.; SWU-B-CC-010030.

##### Distribution.

China (Beijing, Hebei, Henan, Inner Mongolia, Liaoning, Jilin, Tianjin, Shaanxi, Shanxi, Ningxia, Shandong, Jiangsu, Anhui, Hubei (new record), Hunan, Chongqing, Guizhou, Yunnan).

##### Remarks.

**Figure 8.**
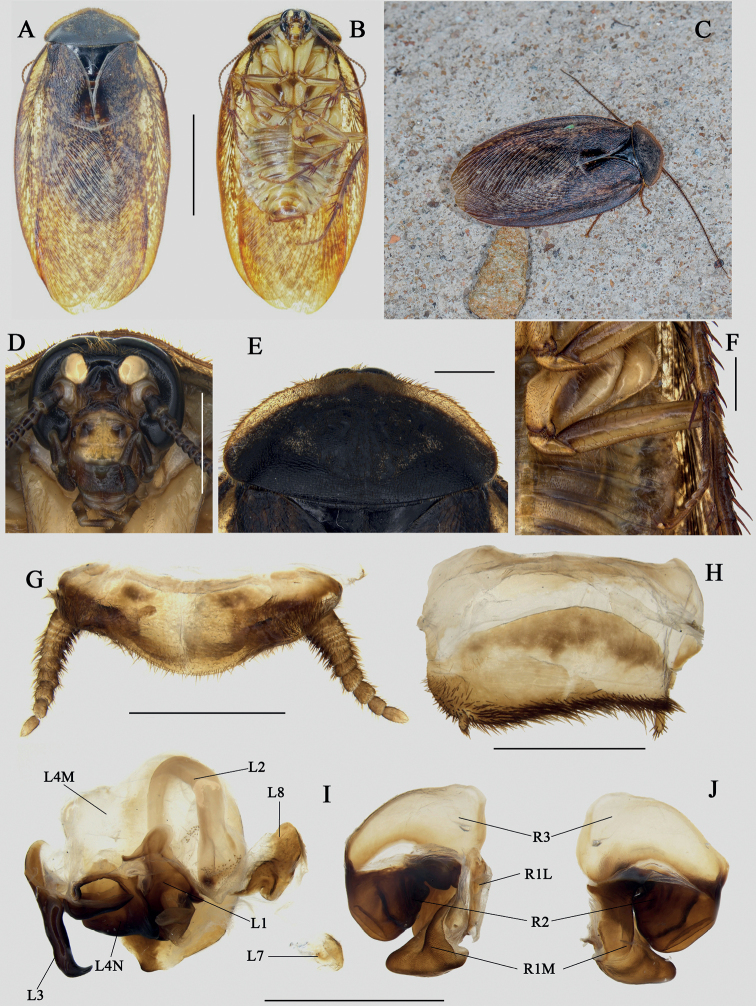
*Eupolyphagasinensis*, male from Sushan Temple, Wuhan City, Hubei Province **A** habitus, dorsal view **B** habitus, ventral view **C** a living male **D** head, ventral view **E** pronotum, dorsal view **F** legs and abdomen (showing dark brown markings), ventral view **G** supra-anal plate, ventral view **H** subgenital plate, ventral view **I** genitalia, dorsal view **J** right phallomere, right-ventral view. Scale bars: 1.0 cm (**A, B**); 0.2 cm (**D–J**). Photographs **C** by Chen-Liang Li.

This species is newly recorded from Hubei Province. This is the most widely distributed *Eupolyphaga* species in China, but in general it is more common in Northern China than in the south. The distribution of this species in south China needs to be further surveyed in the future. The ventral abdomen of this species in males is usually uniformly yellowish white, while a specimen from Wuhan City has dark brown markings on the legs and abdomen (Fig. 8B, F). By sequencing the COI genes of both individuals from Wuhan and northern China (Beijing and Liaoning), we found that the genetic distances between the individuals from Wuhan and north China reached 8.81% (Beijing) to 9.54% (Liaoning); the ABGD analysis treats the Wuhan individual as a separated MOTU from individuals from northern China. Currently only one specimen is available from Wuhan. Therefore, we tentatively regard this variation as an intraspecific difference.

#### ﻿ Eupolyphaga hanae

Taxon classificationAnimaliaBlattodeaCorydiidae

Qiu, Che & Wang, 2018

CFD175AF-E0F6-5265-AE10-7005C6D20A1A

Eupolyphaga hanae Qiu, Che & Wang., 2018: 16; Qiu et al. 2019: 11 (catalogue).

##### Type locality.

“Sichuan Prov., Chengdu City, Dujiangyan Prefectural-Level City, Daguan Town, Puzhao Temple; 770 m.”

##### New material examined.

China · 2 males, 1 female, 10 nymphs, 4 oothecae; Sichuan Prov., Luzhou City, Gulin County, Jianzhu Township, Daheidong Scenic Area, Xiazhai; 899 m; 28°03.50'N, 105°34.70'E, 1 February 2019, Lu Qiu leg.; SWU-B-CC-010031 to 010043 · 1 male, Sichuan Prov., Aba Prefecture, Wenchuan County, Yingxiu Town; 1000 m; 17 September 1983; Xue-Zhong Zhang leg.; SWU-B-CC-010044 · 2 males, Chongqing City, Jiangjin District, Simianshan Mountain, Dawopu; 31 August 2018; Lu Qiu leg.; SWU-B-CC-010045 to 010046 · 1 male, Chongqing City, Jiangjin District, Simianshan Mountain; 22 October 2006, Wei-Wei Zhang leg.; SWU-B-CC-010047 · 1 male, Chongqing City, Jiangjin District, Simianshan Mountain; August 2018; De-Yao Zhou leg.; SWU-B-CC-010048.

##### Remarks.

This species is mainly distributed in Sichuan, Chongqing. We obtained COI sequences of this species from various localities (Simianshan Mountain and Jinyunshan Mountain in Chongqing, Shehong, Dujiangyan and Gulin in Sichuan). Genetic distances ranged from 2.02–6.12% between samples from these localities (Suppl. material 1).

#### ﻿ Eupolyphaga robusta

Taxon classificationAnimaliaBlattodeaCorydiidae

Qiu, Che & Wang, 2018

394932AF-A5AC-5001-8197-3939070ECF5A

Figs 4A, C , 9

Eupolyphaga robusta Qiu, Che & Wang., 2018: 19; Qiu et al. 2019: 11 (catalogue).

##### Type locality.

“Sichuan Prov., Aba Prefecture, Wenchuan County; 1100 m.”

##### New material examined.

China · 2 males, 1 female, 1 nymph, 6 oothecae; Sichuan Prov., Aba Prefecture, Maoxian County, Nanxin Town, Miancu Village; 1518 m; 31°35.73'N, 103°44.97'E; 7 August 2019; Zong-Qing Wang, Lu Qiu, Wei Han, Huan-Yu Ren leg.; SWU-B-CC-010049 to 010052 · 1 nymph, 1 ootheca; Sichuan Prov., Aba Prefecture, Maoxian County, Nanxin Town, Miancu Village; 1503 m; 31°35.77'N, 103°45.00'E; 2 October 2019; Lu Qiu, Hao Xu, Zhi-Teng Chen leg.; SWU-B-CC-010053 · 1 male, 1 female, 10 oothecae; Sichuan Prov., Aba Prefecture, Maoxian County, Xiaomiao Mountain; 1650 m; 31°40.85'N, 103°51.33'E; 6 August 2019; Lu Qiu, Wei Han, Huan-Yu Ren leg.; SWU-B-CC-010054 to 010055 · 1 male, 1 female, 1 nymph, 4 oothecae; Sichuan Prov., Aba Prefecture, 013 Township road near Wenchuan County; 1473 m; 31°28.63'N, 103°35.90'E; Zong-Qing Wang, Lu Qiu, Wei Han, Huan-Yu Ren leg.; SWU-B-CC-010056 to 010057 · 2 males, 6 females, 2 oothecae; Sichuan Prov., Aba Prefecture, Wenchuan County, Miansi Town; 29 March 2020; Jian-Yue Qiu leg.; SWU-B-CC-010058 to 010065.

##### Supplementary description.

**Male**: Tegmina almost black, with a few white maculae, some individuals have more and larger maculae that are randomly distributed on tegmina. Abdomen orange or black, if black, the terminal two segments dimly yellowish.

**Figure 9.**
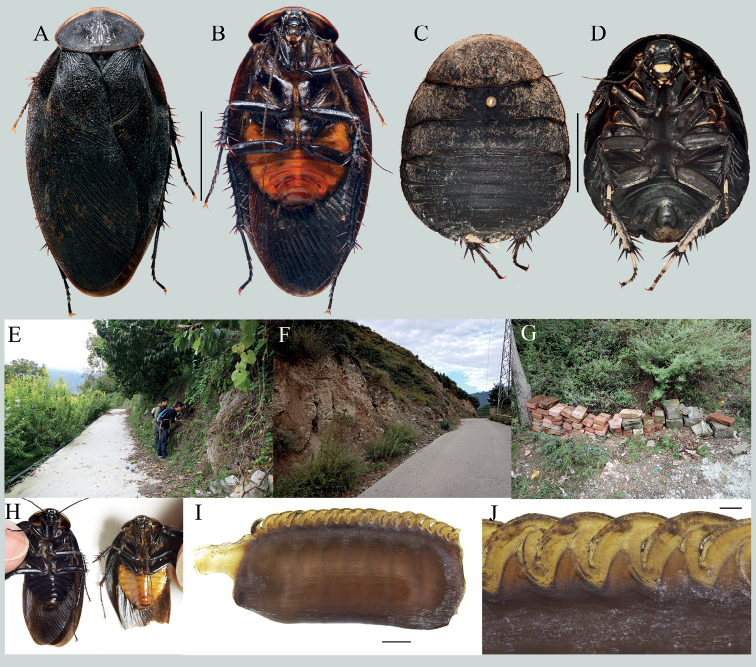
*Eupolyphagarobusta* from Minjiang River basin **A** male, dorsal view **B** male, ventral view **C** female, dorsal view **D** female, ventral view **E–G** habitats of *Eupolyphagarobusta*, Maoxian County, Sichuan Province **H** the variation of the abdominal coloration in males **I** ootheca, lateral view **J** serrations of ootheca. Scale bars: 1.0 cm (**A–D**); 0.1 cm (**I**); 0.01 cm (**J**). Photographs **E–H** by LQ.

**Female**: Body length 29.1–32.5 mm, body width 20.5–22.5 mm. Body uniformly black (Fig. 9C, D). Antennal sockets, ocelli, ante-clypeus white. The base and end of labrum white, the middle black. Ocelli small, nearly triangular. Distance between ocelli smaller than distance between antennal sockets, interocular space wide. Pullivi and arolia absent. Hind margin of supra-anal plate protruded, medially with a large incision (Fig. 9C, D).

**Nymph**: Similar to female, blackish brown.

**Ootheca**: Yellowish brown. Surface with shallow longitudinal lines. Serrations on the keel large and curved, compactly arranged. Respiratory canals indistinct (Fig. 9I, J).

##### Natural history.

This species is distributed in the Minjiang River basin of Sichuan, which is a typical arid river valley. It can be found in multiple habitats, such as in soil, under abandoned bricks or rocks, or inside dead wood.

##### Remarks.

This species was originally described based on a single male specimen collected in 1983. The holotype is dark brown, the last two segments of abdomen are yellowish white, and its vertex has a small yellowish spot. During two expeditions in Minjiang River basin in 2019, we obtained numerous specimens of this species. We found that the fresh samples of the males are black rather than dark brown. The maculae on tegmina show some variation, most male individuals are black, with sparse whitish maculae, but there are individuals with larger maculae. We also found that most of the newly collected individuals differ from the holotype by the coloration of abdomen. Most males have a uniformly orange abdomen, while the specimens from Miansi Town have the same abdominal color as the holotype, i.e., the apical two segments of abdomen are yellowish, the remaining portions are black. Among the newly collected male specimens, no individual was found with a yellow spot at vertex, which indicates the yellow spot at vertex in the holotype may be a variation or aberration.

We sequenced the COI marker from eight samples and found that the genetic distances between samples from Miansi Town (male with black abdomen) and samples from other localities (males with orange abdomens) are somewhat distant (3.29–3.57%), while the genetic distances between samples with orange abdomen are much lower (0.15–0.47%) (Suppl. material 1).

#### ﻿ Eupolyphaga hupingensis

Taxon classificationAnimaliaBlattodeaCorydiidae

Qiu, Che & Wang, 2018

1E2C4264-2B26-59A0-AD2E-331C7872917B

Figs 10A–D , 11

Eupolyphaga hupingensis Qiu, Che & Wang., 2018: 18; Qiu et al. 2019: 11 (catalogue).

##### Type locality.

“China: Hunan: Daling Village, Hupingshan Town, Shimen County”, 444 m.

##### New material examined.

China · 1 male, 2 females, 1 ootheca; Hunan Prov., Zhangjiajie City, Sangzhi County, Badagongshan Nature Reserve, Tianpingshan Mountain; 1400 m; 29°46.99'N, 110°05.44'E; 30–31 July 2019; Hao Xu, Jian-Yue Qiu leg.; SWU-B-CC-010066 to 010068 · 1 male; Anhui Prov., Jinzhai County, Mazongling; 580 m; 2 August 2018, Yu-Chen Zheng leg.; SWU-B-CC-010069.

##### Supplementary description.

**Figure 10.**
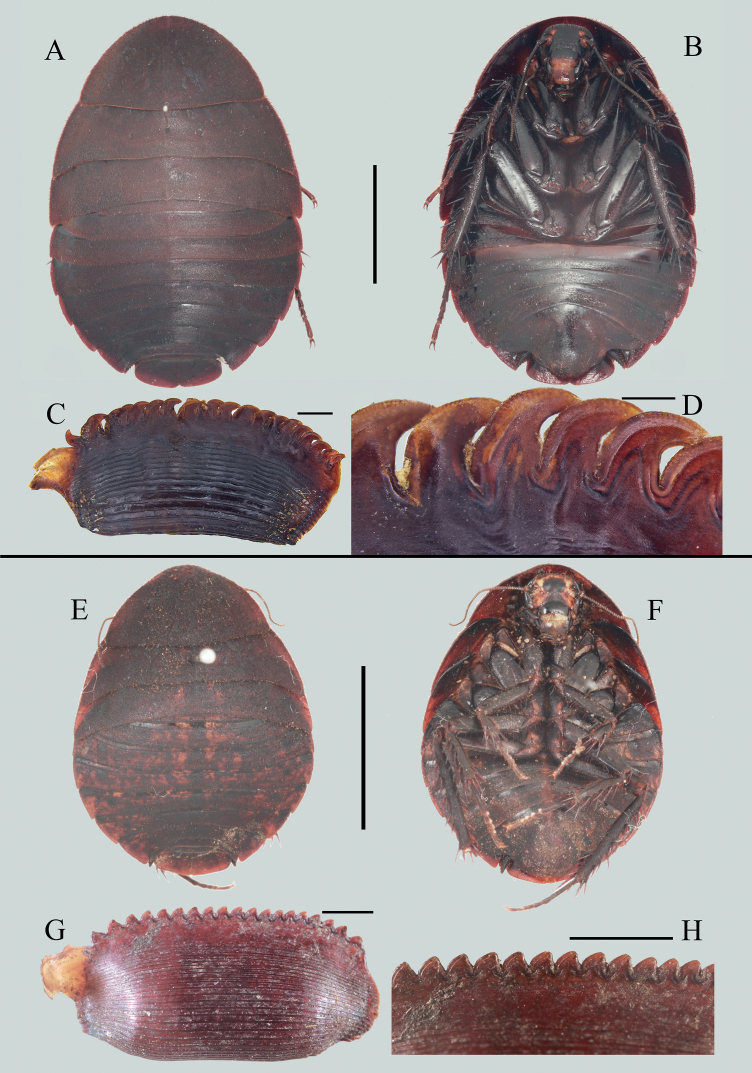
*Eupolyphagahupingensis* from Mt. Badagongshan, Hunan Province (**A–D**) and *Eupolyphagadongi* from Mt. Gaoligongshan, Baoshan City, Yunnan Province (**E–H**). **A** female, dorsal view **B** female, ventral view **C** ootheca, lateral view **D** serrations of ootheca **E** female, dorsal view **F** female, ventral view **G** ootheca, lateral view **H** serrations of ootheca. Scale bars: 1.0 cm (**A, B, E, F**); 0.2 cm (**C, G**); 0.05 cm (**D, H**).

**Female**: Body length 32.3–41.0 mm, body width 21.5–23.7 mm. Body uniformly dark reddish brown. Antennal sockets, ocelli, ante-clypeus, and labrum white. Ocelli large. Distance between ocelli almost equal to interocular space, and slightly narrower than distance between antennal sockets. Arolia and pulvilli absent. Posterior margin of supra-anal plate protruded, medially with a large incision (Fig. 10A, B).

**Nymph.** Similar to female, blackish brown.

**Ootheca.** Reddish brown. Longitudinal lines distinct and evenly arranged (Fig. 10C). Serrations of keel large, strongly curved, the space between the nearest two serrations compact, each serration with weak respiratory canals (Fig. 10D).

##### Distribution.

China: Hunan, Anhui (new record).

##### Remarks.

**Figure 11.**
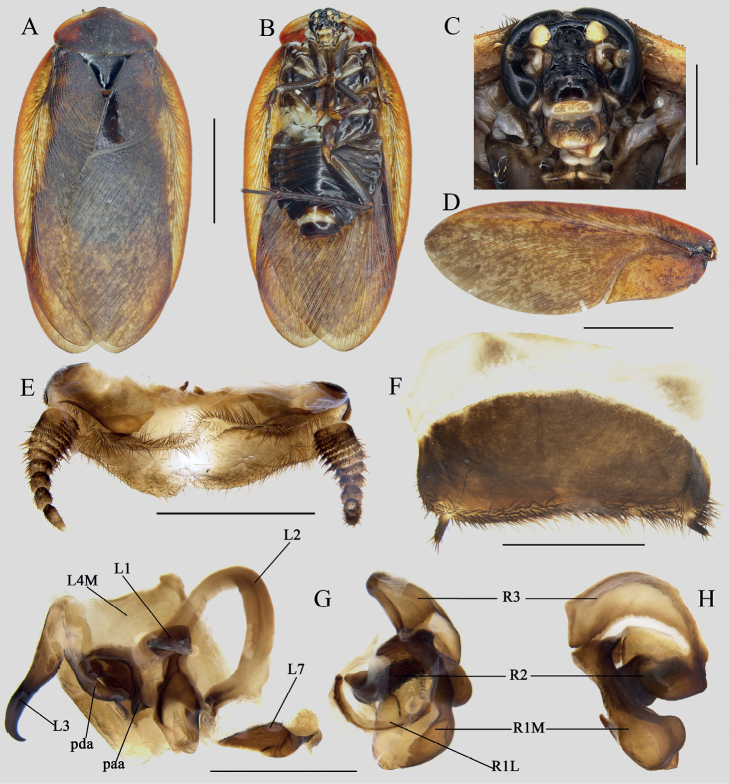
*Eupolyphagahupingensis*, male from Mazongling, Jinzhai County, Anhui Province **A** habitus, dorsal view **B** habitus, ventral view **C** head, ventral view **D** tegmen, ventral view **E** supra-anal plate, ventral view **F** subgenital plate, ventral view **G** genitalia, dorsal view **H** right phallomere, right-ventral view. Scale bars: 1.0 cm (**A, B, D**); 0.2 cm (**C, G, H**); 0.1 cm (**E, F**).

The individual from Jinzhai County (North Anhui) is almost morphologically identical to other specimens of *E.hupingensis*, only the color of its clypeus and labrum, abdomen and legs are lighter than those of holotype (Fig. 11B, C), and styli are slender (Fig. 11F). However, the COI genetic distance between individuals from Jinzhai and Shimen County (North Hunan) reached 8.21%. Because only one specimen is available from Jinzhai, we temporarily regard it as intraspecific variation and look forward to obtaining more samples for further investigation.

#### ﻿ Eupolyphaga dongi

Taxon classificationAnimaliaBlattodeaCorydiidae

Qiu, Che & Wang, 2018

C670D8EF-2738-54AA-B694-AF036A4F7B7C

Fig. 10E–H

Eupolyphaga dongi Qiu, Che & Wang, 2018: 52; Qiu et al. 2019: 11 (checklist).

##### Type locality.

“Yunnan Prov., Baoshan City, Longyang District, Nankang Village; 1980 m.”

##### New material examined.

China · 1 male; Yunnan Prov., Baoshan City, Mt. Gaoligongshan Nature Reserve, Baihualing; 13 April 2017; Zhi-Wei Dong leg.; SWU-B-CC-010070 · 2 females, 1 nymph, 8 oothecae; Yunnan Prov., Baoshan City, Mt. Gaoligongshan Nature Reserve, Baihualing; 20–21 June 2020; Lu Qiu, Jin-Lin Liu leg.; SWU-B-CC-010071 to 010073.

##### Supplemental description.

**Female**: Body length 20.1–21.2 mm, body width 14.6–14.9 mm. Body dark brown, with small fuzzy markings (Fig. 10E). Face with a large black spot, vertex and antennal sockets forming a pale-colored frame enclosing the large black spot. Ocelli degraded and not distinct. Tibial spines reddish brown, pulvilli present, arolia absent. Abdomen with some inconspicuous pale-colored markings and a pale-colored midline. The posterior edge of the supra-anal plate protruded, median emargination small. Posterior margin of subgenital plate wide, with an inconspicuous central shallow concavity (Fig. 10E, F).

**Nymphs**: Similar to female.

**Ootheca**: Reddish brown. Surface with obvious longitudinal lines (Fig. 10G). Serrations short and thick, nearly triangular, terminus blunt, without respiratory canals (Fig. 10H).

##### Natural history.

This species can be found in dry soil under huge rocks in the forest of Baihualing.

## ﻿Discussion

In recent years, ABGD has been successfully applied in the species delimitation of some Blattodea genera (e.g., *Cryptocercus*, Bai et al. 2018; Ectobiidae, Che et al. 2017; *Margattea*, He et al. 2021; *Anaplecta*, Deng et al. 2020, Zhu et al. 2022). So far, species of Corydioidea have rarely been studied by molecular species delimitation methods (Trotter et al. 2017). In this study, the ABGD method was used in support of morphological species delimitation of *Eupolyphaga*, and the results were consistent except for *E.sinensis* and *E.hupingensis*.

Samples of *E.sinensis* from three different localities (Beijing, Liaoning, and Wuhan) were divided into two MOTUs. Compared to the genetic distance between individuals from Beijing and Liaoning (4.76%), the individual from Wuhan was significantly more genetically distant from the first two (8.81% and 9.54%, respectively) (Suppl. material 1). Although the color of the abdomen and legs of the Wuhan individual (Fig. 8A–C) differs from those of other *E.sinensis*, its other characteristics (including male genitalia) exactly coincide with those characteristics of *E.sinensis*. For *E.hupingensis*, which was also divided into two MOTUs by the ABGD method, the genetic distance between Anhui and Hunan samples reached 8.21%. However, the dense maculae on the tegmina, the brown-black but slightly yellow abdomen on the last two segments (Fig. 11A, B, D) and the robust L3 (Fig. 11G) suggest that these two MOTUs belong to the same species. Alternatively, the delimitation results of ABGD of these two species (*E.sinensis* and *E.hupingensis*) could suggest that there may be cryptic species. Since only one male sample from Wuhan and Jinzhai were collected, we temporarily regard them as intraspecific variation and reserve for obtaining more samples for more in-depth research.

Not all species have as unusually large intraspecific genetic distances as *E.sinensis* and *E.hupingensis*. For *E.hanae*, *E.yunnanensis*, and *E.robusta*, samples from different geographic populations were not significantly different morphologically, and their genetic distances (*E.hanae*, 2.02%–6.12%; *E.yunnanensis*, 0.30%–2.01%; *E.robusta*, 0.15%–3.57%, Suppl. material 1) were much lower than that with *E.sinensis* and *E.hupingensis*.

The genus *Eupolyphaga* is widely distributed in China, especially in the mountainous regions of Western China. It is still under-researched due to its secretive habits, which make it not easily detected in the natural environment. The low dispersal ability and the habit of inhabiting only in specific micro-habitats may lead to their long-term geographical isolation (Qiu et al. 2018), and their homogeneous habitat (dry soil and humus) is hardly affected by external environment changes. These two facts may lead to genotypic divergence over phenotypic divergence, and ultimately cause relatively large genetic distances between species without much change in external morphology. We speculate there are still a large number of undiscovered species, and a more extensive field survey of the vast unexplored mountainous areas in Western China is still needed. In the future, studies of this genus can be more comprehensive by incorporating female external genitalia and other characters.

## Supplementary Material

XML Treatment for Eupolyphaga miracidia

XML Treatment for Eupolyphaga udenostyla

XML Treatment for Eupolyphaga reducta

XML Treatment for Eupolyphaga simila

XML Treatment for Eupolyphaga sinensis

XML Treatment for Eupolyphaga hanae

XML Treatment for Eupolyphaga robusta

XML Treatment for Eupolyphaga hupingensis

XML Treatment for Eupolyphaga dongi

## Supplementary materials

Supplementary material 1 Table S1 Data type Genetic distance. Explanation note Genetic distances between COI gene of 45 *Eupolyphaga* species. https://binary.pensoft.net/file/738842This dataset is made available under the Open Database License (http://opendatacommons.org/licenses/odbl/1.0/). The Open Database License (ODbL) is a license agreement intended to allow users to freely share, modify, and use this Dataset while maintaining this same freedom for others, provided that the original source and author(s) are credited.Author: Wei Han, Lu Qiu, Jing Zhu, Zong-Qing Wang, Yan-Li Che
